# Baseline Characterization of Dengue Epidemiology in Yogyakarta City, Indonesia, before a Randomized Controlled Trial of *Wolbachia* for Arboviral Disease Control

**DOI:** 10.4269/ajtmh.18-0315

**Published:** 2018-09-17

**Authors:** Citra Indriani, Riris A. Ahmad, Bayu S. Wiratama, Eggi Arguni, Endah Supriyati, R. Tedjo Sasmono, Fita Yulia Kisworini, Peter A. Ryan, Scott L. O’Neill, Cameron P. Simmons, Adi Utarini, Katherine L. Anders

**Affiliations:** 1 Centre of Tropical Medicine, Faculty of Medicine, Public Health and Nursing, Universitas Gadjah Mada, Yogyakarta, Indonesia;; 2 Department of Epidemiology, Biostatistics and Population Health, Faculty of Medicine, Public Health and Nursing, Universitas Gadjah Mada, Yogyakarta, Indonesia;; 3 Department of Pediatrics, Faculty of Medicine, Public Health and Nursing, Universitas Gadjah Mada, Yogyakarta, Indonesia;; 4 Eijkman Institute for Molecular Biology, Jakarta, Indonesia;; 5 Yogyakarta City Health Office, Yogyakarta, Indonesia;; 6 Institute of Vector Borne Disease, Monash University, Melbourne, Australia;; 7 Wellcome Trust Major Overseas Programme, Oxford University Clinical Research Unit, Hospital for Tropical Diseases, Ho Chi Minh City, Vietnam;; 8 Department of Health Policy and Management, Faculty of Medicine, Public Health and Nursing, Universitas Gadjah Mada, Yogyakarta, Indonesia

## Abstract

Dengue is endemic in Indonesia. Here, we describe the epidemiology of dengue in the city of Yogyakarta, Central Java, as a prelude to implementation of a cluster-randomized trial of *Wolbachia* for the biocontrol of arboviral transmission. Surveillance records from 2006 to 2016 demonstrate seasonal oscillations of dengue incidence with varying magnitude. Two lines of evidence demonstrate a high force of infection; the hospitalized case burden of patients diagnosed with dengue hemorrhagic fever or dengue shock syndrome over the last decade consisted predominantly of children/adolescents, and a serosurvey of 314 healthy children aged 1–10 years found 68% possessed dengue virus–neutralizing antibodies. Finally, a mobility survey indicated children aged 1–10 years, and particularly 1–5 year-olds, spent most of their daytime hours at home. These findings inform the design of clinical trials to measure the impact of novel vector control methods such as *Wolbachia* introgression into *Aedes aegypti* mosquitoes, by providing baseline data on disease incidence and identifying subpopulations for recruitment into prospective studies of dengue virus infection and disease. The mobility survey findings indicate that in cluster trials of interventions applied at the community level, young children can reasonably be expected to spend most of their exposure time, in epidemiological terms, within the treatment arm to which they were randomized.

## INTRODUCTION

Dengue is an acute mosquito-borne viral infection. It is considered a major public health problem in many tropical and subtropical regions of the world, with an estimated 50–100 million symptomatic infections occurring each year.^1,2^ In Indonesia, the first case of dengue hemorrhagic fever (DHF) was reported in 1968.^3^ Dengue has since emerged as a major public health problem and the country carries the highest case burden in South East Asia.^2–5^ Dengue is a notifiable disease within the national surveillance system, coordinated by the Indonesian Ministry of Health, with passive case reporting of DHF and dengue shock syndrome (DSS) cases from both government and private hospitals.^6^

Dengue cases have been reported from all provinces of Indonesia since 1994. In 2009, 11/32 provinces (33%) were considered as high-risk areas, with an incidence rate ≥ 55 cases per 100,000 population.^4^ The notified dengue incidence rate in Yogyakarta Province has increased since 2005, from 20 cases per 100,000 to more than 55 cases per 100,000 from 2006 onward. The Indonesian Ministry of Health has identified Yogyakarta Province as one of the 10 provinces most affected by dengue each year in the last three decades.^4,7^

The continued rise in dengue case burden in Indonesia and worldwide over recent decades highlights the difficulty of achieving a large and sustained impact on transmission with traditional methods of vector control, at least within the financial and resource constraints faced by public health programs in many tropical countries. The first dengue vaccine candidate, Dengvaxia (Sanofi-Pasteur), has been licensed for use in several countries since 2016, including Indonesia, but with a limited target population because of its complex efficacy and safety profile.^8^ There remains a well-recognized need for both improved approaches to dengue prevention and control^9^ and for more rigorous evaluation of existing and novel methods^10,11^ to provide a robust evidence base to inform dengue control programs.

Local data on dengue disease incidence and distribution are critical both to inform the feasibility and rational design of efficacy trials of dengue preventive interventions and later to evaluate cost-effectiveness and to target implementation.^1,12^ Time series of case notifications give an indication of the burden of disease (albeit with imperfect sensitivity and specificity), spatial and temporal trends in disease incidence, and the subpopulations most affected. Seroprevalence data can be used to infer age-specific transmission rates and median age at first infection. An understanding of population mobility is important for cluster-randomized trials (CRTs) where the intervention is allocated at the community level rather than at the individual-level, as is commonly the case for vector control studies.^13^ High mobility and/or small cluster size will lead to greater contamination between the treatment arms, thus reducing the statistical power of the study to detect an intervention effect. Understanding local spatial and temporal heterogeneity in dengue transmission is also important for calculating sample size requirements for CRTs, with increased spatial or temporal heterogeneity necessitating a larger number of clusters and/or longer study period.^12^

Here, we present data from a combination of retrospective and prospective studies conducted in Yogyakarta that aimed to characterize the epidemiology of dengue in this setting and quantify the mobility of “at-risk” population groups, to inform the feasibility and design of future intervention studies.

## METHODS

### Setting and location.

**Figure 1.**
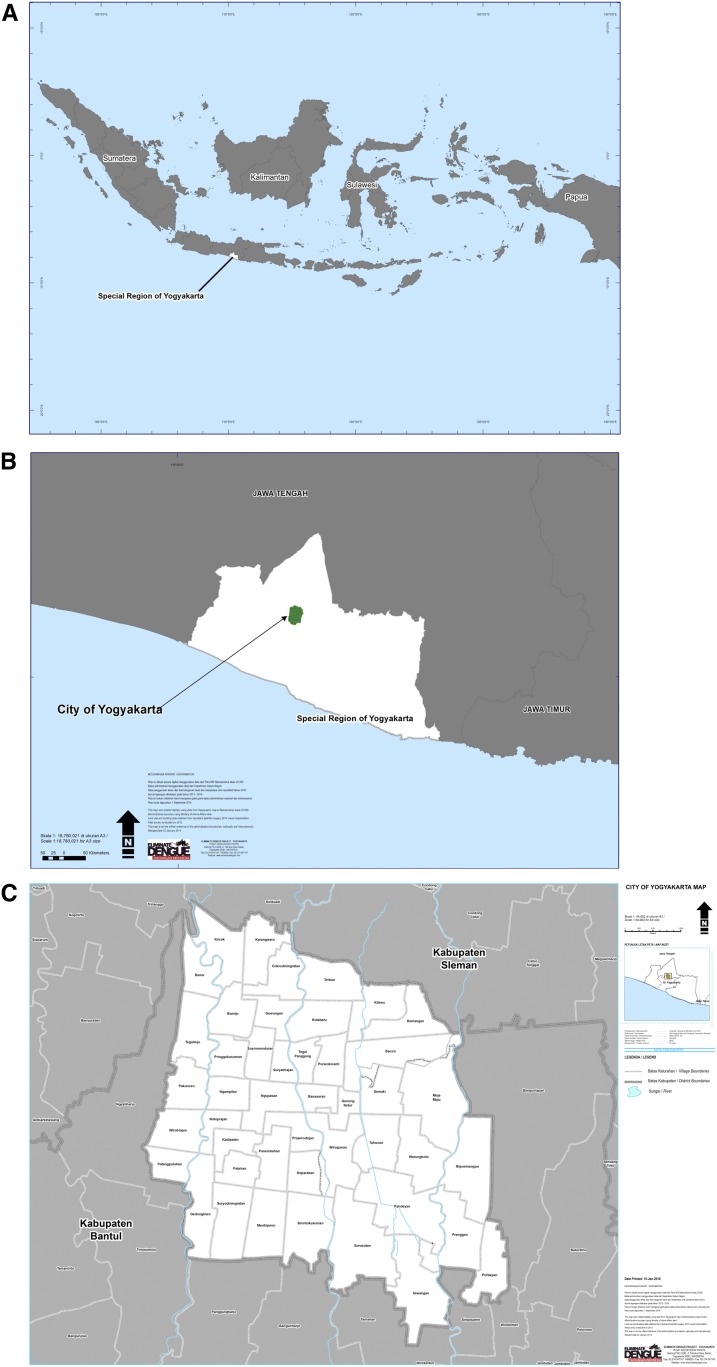
Geographical location of Yogyakarta city in Indonesia (**A**) and in Special Region of Yogyakarta (**B**), with details of administrative boundaries (**C**). This figure appears in color at www.ajtmh.org.

Yogyakarta city (Figure 1) is an urban center in south-central Java, with a population of 417,744 residents in 2016 living in an area of 32.5 square kilometers. The city is the capital of Yogyakarta Province (Special Region) and has administrative subdivisions of 14 districts and 45 villages (kelurahan).^14^ There is a pronounced rainy season from November to May.

### Dengue surveillance system and case definition.

De-identified data on the number of dengue cases reported to the routine dengue surveillance system were obtained from the Yogyakarta City Health Office, aggregated by month and kelurahan of residence, for 11 years from January 2006 to December 2016. Individual data on the age of dengue cases notified during 9 years (2006–2014) were also obtained (age-stratified data were not available for 2015–2016). Notified dengue cases include only those classified as DHF or DSS, as per the Ministry of Health guidelines published in 2005 and revised in 2011, which rely on a clinical case definition. Dengue hemorrhagic fever and DSS were defined according to the 1997 WHO criteria.^15^ Notifications include only hospitalized dengue cases, not those seen as outpatients. We used annual population data (from the Yogyakarta Office of Statistics) for calculating per capita incidence rate.

### Seroprevalence study and mobility survey.

#### Sampling strategy.

Two-stage cluster sampling^16^ was used to randomly select children aged 1–10 years for enrolment in a cross-sectional seroprevalence survey and mobility survey, in May–June 2016. Sampling units for the first stage were the lowest level administrative unit (Rukun Tetangga [RT]), each of which has approximately 50 households. A list of all 2,529 RTs in Yogyakarta city was obtained from the municipality civil registry office, and 50 RTs were randomly selected using random number generation. In each selected RT, a household number list was obtained from the RT leader and used as the second sampling frame to select 10 households. All children aged 1–10 years who were permanently residing in the selected households were invited to participate, with an aim of achieving a balanced distribution of participants across 2-year age bands. If there were no eligible children of the required age group in the selected household, or if the family declined to participate, the nearest house was approached until at least one child was enrolled. Enrolment was extended to an additional randomly selected 16 RTs to give a total of 66, to achieve the target sample size and age distribution of enrolled children, as in some RTs fewer than 10 children could be enrolled.

#### Sample size.

In calculating the target sample size for the seroprevalence survey, we assumed that the overall seroprevalence of dengue virus (DENV)-reactive IgG in the study population aged 1–10 years is 50%. We further assume an intracluster (i.e., within-RT) correlation coefficient (ρ) of 0.2. The factor by which the variance is inflated because of the cluster sampling design (the design effect), assuming 50 primary sampling units (RTs), is therefore 2.0, resulting in a standard error of 0.04.^16^ This translates to a precision of ±8% around the seroprevalence estimate, with a sample size of 300, sampled as described earlier.

Because the planned analyses of mobility data were descriptive and exploratory in nature, and we had no baseline data from which to estimate the distribution of respondents’ mobility, we did not attempt a formal sample size calculation for the mobility survey. Instead, we pragmatically applied a target sample size of 500 and invited all participants in the mobility study to enroll also in the seroprevalence survey, with an expectation that at least 300 of those who consented to the mobility survey would also consent to a blood draw for the seroprevalence survey. Sampling was age-stratified to achieve equal numbers of participants in each of the five 2-year age bands.

#### Data collection.

At the selected households with children aged 1–10 years, a trained interviewer explained the study in detail and obtained written consent from a parent or guardian. Data on children’s mobility were collected using a combination of a structured face-to-face interview and a prospective 7-day travel diary, both completed by the household member who spent most time with the child each day. At enrolment, the interviewer recorded data on household sociodemographics, the child’s regular activities outside the home and time spent with them, and the location of the child’s school or preschool and other routinely visited places. Tablets were used to enter interview data directly into a structured electronic data capture form using a customizable data capture tool (iFormBuilder; Zerion Software, Herndon, VA). Data were uploaded to a central database at the end of each day and were reviewed and validated on a daily basis. The interviewer contacted the respondent and parent or guardian each day for the next 7 days, either by phone or house visit, to record in a structured travel diary of all places visited by the child during 7 days, including the time of arrival and departure, the location type, name, and address if known. At day 8, the interviewer returned to review with the child’s guardian all the places recorded in the diary. During the review, the child’s guardian was asked to locate each unique place visited for at least 1 hour, on a tablet-based digital map (Map Coordinates App v4, Soft Stack Dev, Arad, Romania; play.google.com/store/apps/details?id=sands.mapCoordinates.android), with the assistance of the interviewer. The coordinates of each unique location were transferred directly into the travel history data capture tool.

Separate informed written consent was obtained for participation in the seroprevalence study, although the study population was drawn from the mobility study participants. After signing the informed consent, one 2 mL whole blood specimen was drawn into an EDTA tube. Labeled specimens were kept in a cool box before being transported to the Eliminate Dengue Yogyakarta diagnostic laboratory each day. Data on sociodemographics of the child (gender and age) and a unique identifying number were recorded.

#### Laboratory investigation.

Blood samples were centrifuged and plasma stored at −20°C. Plasma samples were batch tested for DENV-reactive IgG using the Panbio Dengue IgG indirect enzyme-linked immunosorbent assay (ELISA) (Alere, Australia) in the EDP-Yogyakarta diagnostic laboratory, as per manufacturer’s instructions. An age-stratified random sample of specimens positive in IgG indirect ELISA and a smaller number of negative specimens were subsequently tested for DENV serotype-specific neutralizing antibodies by the Plaque Reduction Neutralization Test (PRNT_50_), at the Eijkman Institute Indonesia, to confirm the presence of DENV-neutralizing antibodies. The PRNT_50_ assay was performed using materials and methods as described by Timiryasova et al.^17^ A titer of ≥ 40 was used to define a positive result in the PRNT_50_ assay, for all serotypes.

#### Data analysis.

Data from the prospective travel diaries were aggregated to calculate the total duration of time spent at each unique location over a week, for each respondent. The distance of each visited location from home was calculated using the geodist package in Stata (version 14; StataCorp LP, College Station, TX). The proportion of each respondent’s daytime hours spent at home and at increasing distances from home was calculated by aggregating their hours spent at all locations within each distance interval (100 m bins between 0 and 2 km from home), and dividing by their total aggregate hours documented in the travel diary. Summary statistics were produced within each age group (preschool age: 1–5 years and school age: 6–10 years), and the Wilcoxon rank-sum nonparametric test was used to compare the distribution of individual participants’ proportion of time spent at home, and within 500 m or 1 km of home, between age groups.

Seroprevalence was calculated directly as the proportion of total and age-stratified participants with detectable DENV-reactive IgG, with a binomial 95% confidence interval based on the normal approximation.

#### Ethical considerations.

Ethical approval for the mobility study and seroprevalence survey was obtained from the Faculty of Medicine, Public Health and Nursing, Universitas Gadjah Mada Ethical Committee, with approval number KE/FK/332/EC. Written informed consent to participate in the mobility study and/or seroprevalence survey was obtained from a parent/guardian of all participants. A unique identification number was used for all data and specimen collection.

### Spatial analysis.

Maps of dengue incidence per 100,000 population for each of the 45 administrative areas (kelurahans) in Yogyakarta city, each year between 2006 and 2016, were produced using ArcGIS (ESRI Software, Redlands, CA). The kelurahan-level annual dengue incidence time series were square root transformed to more closely approximate a normal distribution, and standardized to a mean of zero and standard deviation equal to one. Pairwise correlations between kelurahans’ transformed annual dengue incidence were calculated, and the relationship between these correlation coefficients and the intervening distance between kelurahan centroids was assessed using the nonparametric spline covariance function from the NCF package in R (R 3.4.0; R Foundation for Statistical Computing, Vienna, Austria) with 1,000 bootstraps to generate 95% confidence bands. A Mantel test was used to evaluate the overall spatial correlation in annual dengue incidence over all pairwise inter-kelurahan distances.

## RESULTS

### Notified dengue case incidence in Yogyakarta, 2006–2016.

**Figure 2.**
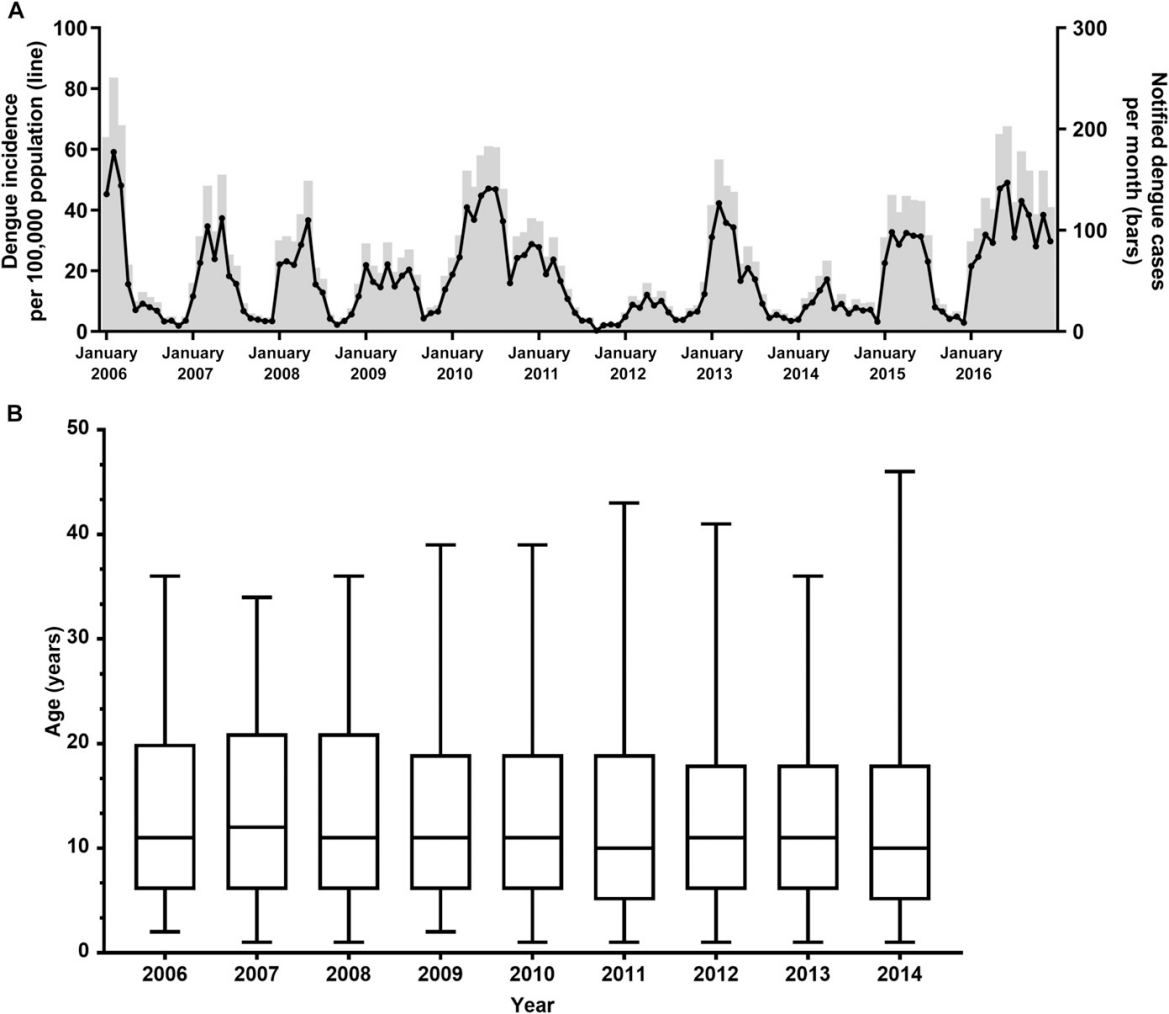
(**A**) Time series of notified dengue cases (bars) and incidence per 100,000 population (line) per month in Yogyakarta city between 2006 and 2016. Data include all hospitalized dengue hemorrhagic fever and dengue shock syndrome cases reported to the passive surveillance system coordinated by the Yogyakarta District Health office. (**B**) Median age (with 5–95 percentiles) of notified dengue cases in Yogyakarta city, 2006–2014.

During the 11-year period, 2006–2016, 9,418 dengue cases were notified in Yogyakarta city. The number of annual case notifications varied from a low of 360 in 2012 (per capita incidence 91/100,000 population) to a high of 1,705 in 2016 (incidence 412/100,000 population), with a mean of 856 cases per year (incidence 211/100,000 population). Peak dengue incidence tended to occur during the first 6 months of the year, although the timing of seasonal peaks varied somewhat year-to-year (Figure 2A). During the peak month each year, between 49 and 251 hospitalized dengue cases were notified corresponding to a monthly incidence of 12–59 per 100,000 population. The overall median age of dengue cases was 11 years(interquartile range [IQR] 6–20 years) and this remained broadly consistent over the 9 years for which age data were available (Figure 2B).

### Spatial patterns in dengue incidence.

**Figure 3.**
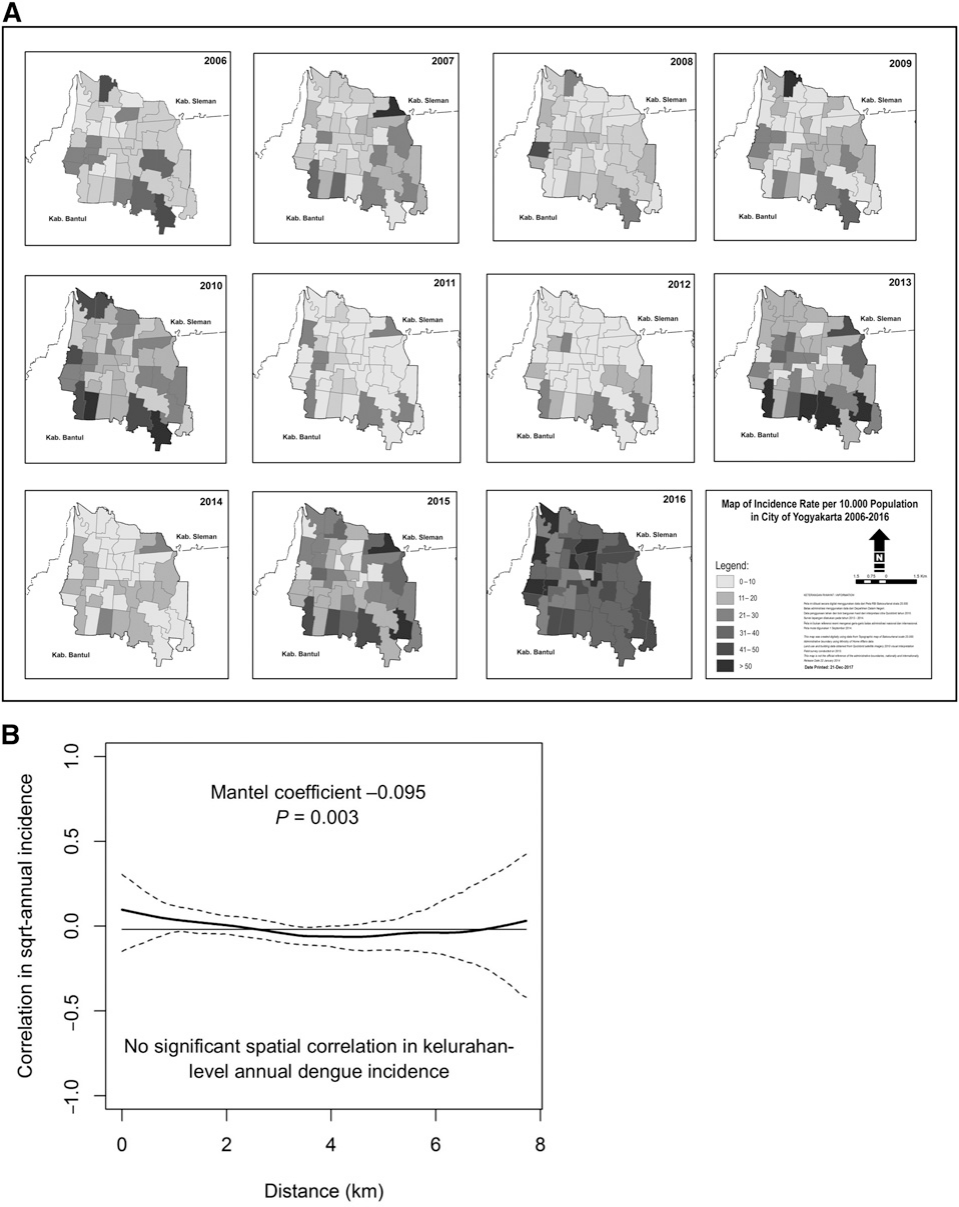
(**A**) Map of 45 administrative areas (kelurahans) in Yogyakarta city, shaded by annual dengue incidence (per 100,000 population) for each year, 2006–2016. (**B**) Nonparametric correlation function shows pairwise correlation (solid line) between 45 kelurahans in Yogyakarta city in their annual dengue incidence (square root transformed and standardized to zero mean and standard deviation of one), as a function of the distance between the centroids of kelurahans. Dashed lines represent 95% confidence intervals, and the horizontal line is the overall correlation in kelurahan-level annual dengue incidence across Yogyakarta city. The Mantel test coefficient reports average spatial dependence in annual dengue incidence over all pairwise inter-kelurahan distances.

Figure 3A shows the annual dengue incidence by administrative area (kelurahan) in Yogyakarta city for each year, 2006–2016. A spatial nonparametric correlation function was used to investigate dengue spatial dependence and demonstrated no significant spatial correlation in annual dengue incidence among the 45 kelurahans of Yogyakarta city (Figure 3B). This was supported by the Mantel test result, with a correlation coefficient very close to zero (Mantel coefficient −0.095; *P* = 0.003).

### Age-stratified DENV seroprevalence in children aged 1–10 years.

**Figure 4.**
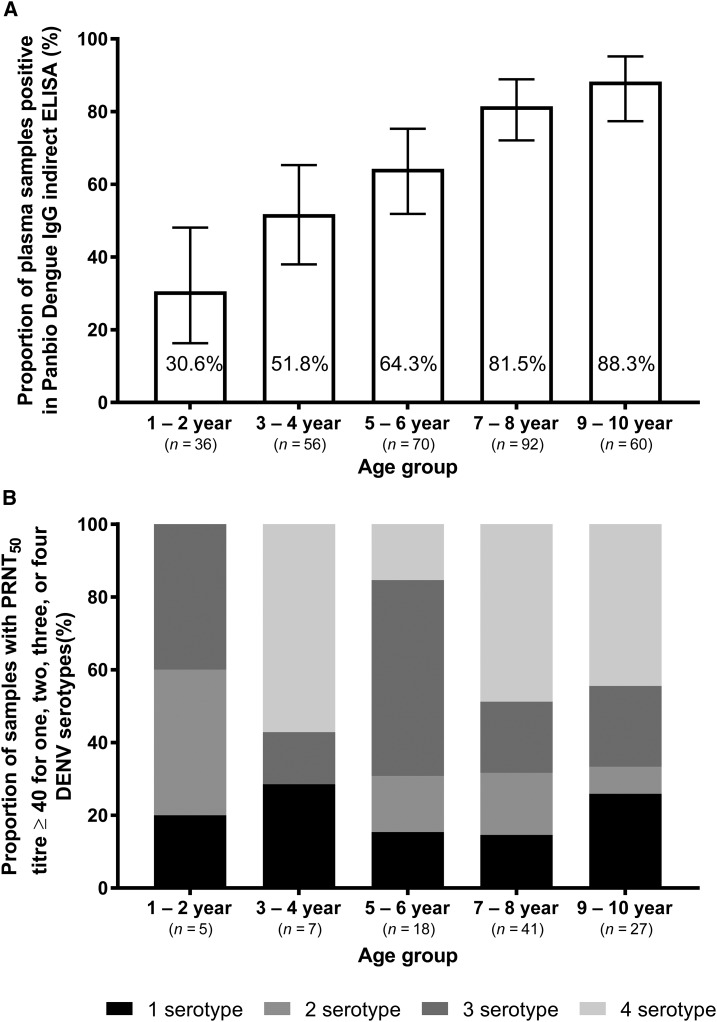
Age-specific dengue seroprevalence among children aged 1–10 years resident in Yogyakarta city, sampled cross-sectionally in May–June 2015. (**A**) Proportion of samples (*N* = 314 total) positive in Panbio dengue IgG indirect ELISA, by 2-year age group, with 95% binomial confidence interval. (**B**) Monotypic and multitypic DENV-neutralizing antibody profiles, determined via PRNT_50_ assay, in a random subset of 98 plasma samples that had previously tested positive in the Panbio IgG indirect ELISA. Three samples positive in IgG indirect ELISA but with PRNT_50_ titers < 40 to all DENV serotypes are not shown.

Understanding age-stratified DENV seroprevalence in Yogyakarta city informs estimates of the intensity of transmission. Given the low median age of notified dengue cases, the seroprevalence survey and mobility survey targeted young children up to 10 years of age. Blood samples were collected from a cross-sectional sample of 314 children aged 1–10 years resident in Yogyakarta, with an age distribution as shown in Figure 4A. The overall seropositivity by Panbio dengue IgG indirect ELISA was 68% (213/314). As expected, seropositivity increased with age (Figure 4A). Among a randomly selected subset of 101 samples positive in IgG indirect ELISA, 98 (97%) had a PRNT_50_ titer ≥ 40 to one or more DENV serotypes. All five randomly selected samples that were negative in IgG indirect ELISA were also below the limit of detection in the PRNT_50_ assay. The PRNT_50_ results demonstrate that the majority of children in all age classes (71–85% of those seropositive in ELISA) possessed multitypic DENV-neutralizing antibody profiles (Figure 4B).

### Mobility of children in Yogyakarta city.

**Figure 5.**
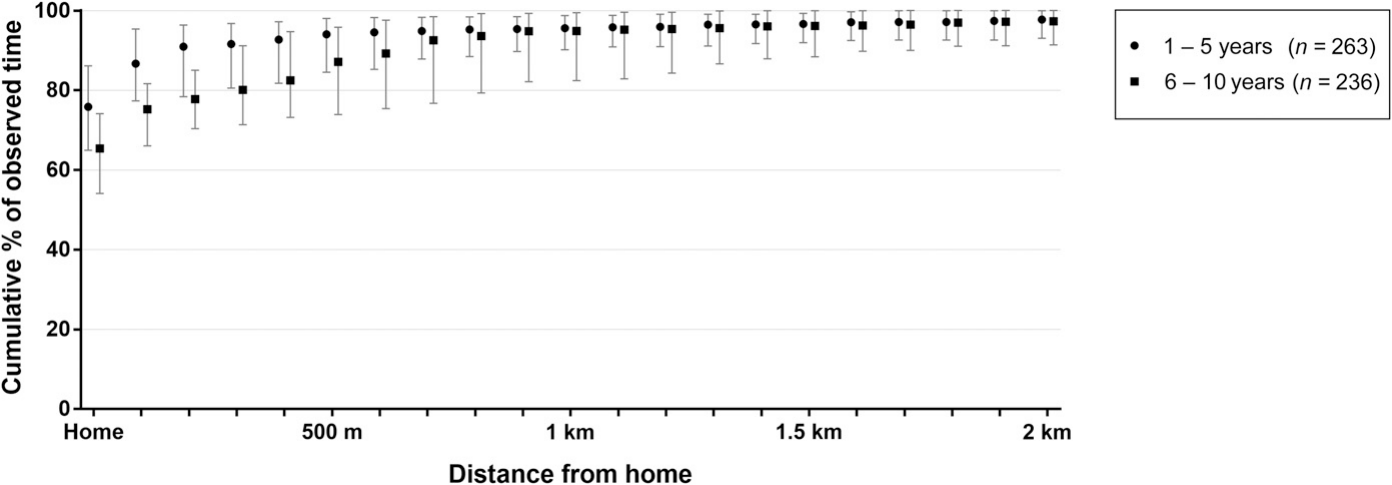
Mobility of children aged 1–10 years in Yogyakarta city, determined by a self-reported travel diary for 7 days (5 am–9 pm) of children’s activity, including weekdays and weekend. The graph shows the median and interquartile range cumulative distribution of the proportion of time (5 am–9 pm aggregated over seven consecutive days) that respondents spent at home and within increasing distances from home, by age group: children aged 1–5 years (preschool) and children aged 6–10 years (school aged).

Understanding human mobility is important for informing the design and analysis of CRTs of dengue preventive interventions, as time spent outside the cluster of residence may reduce the power to measure an intervention effect. A travel diary was completed by 515 children aged 1–10 years, recording their movements between 5 am and 9 pm on seven consecutive days. Figure 5 shows the cumulative distribution of respondents’ time spent at home and at increasing distances from home, as the median and interquartile range of total observed time per respondent, by age group. Younger children aged 1–5 years spent more time at home than older children aged 6–10 years (median [IQR] 76% [65–86%] versus 65% [54–74%]; Wilcoxon rank-sum test *P* < 0.0001). This age difference persisted out to a distance of 500 m from home; among 1- to 5-year-olds, the median (IQR) proportion of respondents’ time spent within 500 m from home was 94% (85–98%), compared with 87% (74–96%) among 6- to 10-year-olds (*P* < 0.0001). At 1 km from home no age difference remained, with children of all ages spending on average 95% of their time within this distance from home. The main secondary locations at which children spent their daytime hours were school for the 6- to 10-year-olds (median 17% of observed hours, IQR 11–22%) and a relative’s house for the 1- to 5-year-olds (median 7% of observed hours, IQR 3–14%).

## DISCUSSION

Here, we illustrate three key aspects of dengue epidemiology in Yogyakarta. First, the historical time series of DHF/DSS incidence confirms annual seasonal outbreaks but with no obvious interannual periodicity or spatial autocorrelation of dengue incidence. Second, findings from a DENV seroprevalence survey indicated that most children have been infected by at least one DENV serotype, and frequently ≥ 1 DENV serotype, before the age of 10 years. Third, a large prospective mobility survey determined that most of the children, and especially young children, spend most of their time at or near their homes. These findings illustrate the public health burden of dengue in Yogyakarta and highlight the limitations of existing vector control strategies to prevent dengue virus transmission. In addition, these results inform the design of clinical trials to measure the impact of novel vector control methods, such as *Wolbachia* introgression into *Aedes aegypti* mosquitoes.^12^

Previous studies have demonstrated that individual dengue cases and administrative area-level dengue incidence^18–24^ are spatially autocorrelated, that is, they cluster together more than expected by chance. Yet, in Yogyakarta, we found no evidence of spatial autocorrelation of kelurahan-level dengue incidence. Possibly, the relatively small size of Yogyakarta city and a high degree of population mixing dissipate any spatial signature. In addition, the dengue incidence data described here are derived from the passive surveillance system, which includes only hospitalized cases diagnosed clinically as DHF or DSS. These data have an imperfect level of sensitivity and specificity with respect to accurate case detection and, thus, may mask a true spatial signature.^25,26^ Estimates of the degree to which data from the routine surveillance system underestimate true dengue incidence, expressed as an expansion (or multiplication) factor, range from 2.3 to 11.5^25–27^ for Indonesia. Regarding imperfect specificity, a prospective trial would need to apply a standardized case definition and laboratory diagnostic algorithm for detecting virologically confirmed symptomatic DENV infections in prospectively enrolled patients and not rely on routine surveillance data. Further research is needed to understand spatial heterogeneity of dengue case incidence because this heterogeneity is an important parameter to consider in the design of cluster-randomized intervention trials.^12^

*Aedes aegypti* prevalence and population sizes are temporally and spatially stable in Yogyakarta.^28^ Existing *Ae. aegypti* mosquito population control activities in Yogyakarta consist of insecticide space spraying, application of larvicide to breeding sites, and education of the population on how to avoid dengue. Yet, it is widely agreed that these methods lack a strong evidence base that supports their application in preventing arboviral disease transmission.^11^ The lack of impact of existing control measures is reflected by the median age of hospitalized dengue cases being ∼11 years for the last decade. Indeed, that ∼52% of children from Yogyakarta aged 3–4 years were seropositive in the DENV IgG indirect ELISA suggests a high force of infection. A recent national serosurvey of Indonesian children in urban environments using the same ELISA technique measured similar frequencies of seropositivity: 33.8% in 1- to 4-year-olds and 65.4% in the 5- to 9-year-olds.^29^ This evidence is entirely consistent with Indonesia being one of the highest dengue burden countries globally.^2^ It also reinforces the challenge of classical mosquito suppression approaches to disease control, that is, a major escalation of mosquito population suppression might delay the timing of an individual’s first infection, but not eliminate lifetime risk. Whether dengue is the only flaviviral disease being transmitted by *Ae. aegypti* in Yogyakarta is unknown, but virologically confirmed Zika cases in Indonesia have been recently reported.^30,31^

Cluster-randomized trials are the gold standard method for measuring the effect of interventions applied at the level of communities. However, cluster trials can be confounded by the movement of study participants away from their assigned cluster, and thus, exposure to their assigned intervention.^13^ Participants may move for school, work, or social reasons and be absent for some or many hours per day. Such movement can dilute (i.e., bias to the null) the measured effect size of a particular intervention, for example, *Wolbachia* introgressed into the local *Ae. aegypti* population to stop DENV transmission. Here, we found that children aged 1–10 years spent > 70% of their time at home in the 7 days before interview. Perhaps not surprisingly, children aged 1–5 years spent more time at home than children aged 6–10 years. These data are reassuring because if children of these ages were participants in a cluster trial, where the intervention was applied at the level of a neighborhood, then these participants would spend most of their time exposed, in epidemiological terms, to the treatment arm to which they were randomized. In addition, adjustment for transient movement of study participants away from their residence in the days leading up to onset of symptomatic dengue can be made at the analysis stage.^12^

In summary, the burden of dengue remains largely unmet by effective disease prevention strategies in Yogyakarta and is emblematic of the wider problem of dengue control in the Asia Pacific. This disease burden is unlikely to be met soon by vaccination programs, given the current concerns over the safety of the only licensed dengue vaccine in seronegative individuals.^8^ New strategies and tools, including novel entomological methods such as *Wolbachia* introgression, urgently need to be tested to tackle the decades-old problem of dengue in Yogyakarta and elsewhere. The baseline epidemiological findings presented here can inform the design of high-quality trials in this setting by providing a baseline estimate of disease incidence and spatial and temporal variability in disease; identifying children and adolescents as an appropriate target group for prospective studies of DENV infection and disease; and demonstrating that this target population of young children has relatively limited mobility, spending the large majority of their time close to home and thus within the treatment area of an intervention delivered at the community level. To this end, the first randomized trial of *Wolbachia* for dengue control commenced in Yogyakarta city in 2017^32^ (ClinicalTrials.gov NCT03055585).
